# Dynamics of immune-checkpoint regulators and inflammatory cytokines and chemokines associated with preterm birth before and after cervical cerclage

**DOI:** 10.3389/fimmu.2026.1731111

**Published:** 2026-06-23

**Authors:** Seri Jeong, Keun-Young Lee, Ga-Hyun Son, Kyong-No Lee, Won Kyong Cho, Hoseong Choi, Jonghyun Lee, Joanne Kwak-Kim

**Affiliations:** 1 Department of Laboratory Medicine, Kangnam Sacred Heart Hospital, Hallym University College of Medicine, Seoul, Republic of Korea; 2 Division of Maternal-Fetal Medicine, Department of Obstetrics and Gynecology, Kangnam Sacred Heart Hospital, Hallym University College of Medicine, Seoul, Republic of Korea; 3 Department of Obstetrics and Gynecology, Chungnam National University College of Medicine, Chungnam National University Hospital, Daejeon, Republic of Korea; 4 Agriculture and Life Sciences Research Institute, Kangwon National University, Chuncheon, Republic of Korea; 5 Biocube System, Inc., Suwon, Republic of Korea; 6 Department of Biostatistics, Epidemiology and Informatics, Perelman School of Medicine, University of Pennsylvania, Philadelphia, PA, United States; 7 Reproductive Medicine and Immunology, Obstetrics and Gynecology, Clinical Sciences Department, Chicago Medical School, Rosalind Franklin University of Medicine and Science, North Chicago, IL, United States

**Keywords:** cerclage, cervical insufficiency, cervix, cytokines, immune-checkpoint regulators, inflammation, preterm birth

## Abstract

**Background:**

Cervical insufficiency (CI), characterized by painless cervical dilation during the second trimester, is a major cause of preterm birth. However, the local microenvironment associated with CI, particularly the roles of immune-checkpoint regulators (ICRs) and inflammatory cytokines and chemokines (ICKs), remains poorly understood. This study aimed to investigate the changes in ICRs and ICKs before and after cervical cerclage, as well as to identify predictors of preterm birth in patients with CI.

**Methods:**

A total of 64 women with CI comprised the study group, and 128 endocervical swab samples were collected before and after cervical cerclage. Participants were categorized according to disease severity, specifically, membrane bulging vs. non-bulging, and delivery outcomes, namely preterm vs. full-term delivery. Seventeen soluble ICRs and 17 ICKs were quantified using a Luminex multiplex assay.

**Results:**

Our results indicated significant differences in immunologic microenvironments before and after cerclage. Among ICRs, BTLA, CD28, GITR, and CD80 (B7-1) levels were significantly decreased after cerclage compared with pre-cerclage levels. In contrast, TIM-3 and PD-L2 were significantly increased, along with twelve other ICKs. Pre-cerclage ICRs and ICKs were generally higher in patients who received emergent cerclage with bulging membranes compared to those without bulging membranes. In particular, pre-cerclage levels of CCL2 (MCP-1), CXCL10 (IP-10), GM-CSF, IL-1α, IL-6, IL-10, and IL-17A were significantly elevated in patients who subsequently experienced preterm birth compared with those who delivered at term. Post-cerclage levels of LAG-3, CTLA-4, CD86 (B7-2), and PD-L2 were significantly lower in the preterm birth group. Among these, pre-cerclage IL-17A was the most significant predictor (odds ratio = 2.3) in the final multivariate model. The combination of pre-cerclage IL-17A and post-cerclage PD-L1 demonstrated excellent predictive performance, with an area under the receiver operating characteristic curve (AUC) of 0.901.

**Conclusion:**

Our study revealed the dynamic changes of ICKs and ICRs following cervical cerclage and identified key markers for preterm birth, including pre-cerclage IL-17A and post-cerclage PD-L1, in patients with CI. By focusing on these components, our work enhances the understanding of the local immune landscape and supports the development of targeted strategies to improve pregnancy outcomes.

## 1 Introduction

Preterm birth, defined as delivery before 37 completed weeks of gestation, affected approximately 13.4 million pregnancies globally in 2020 (1). It remains the leading cause of neonatal mortality and is associated with substantial long-term consequences, including physical disabilities, neurodevelopmental impairments, and socioeconomic challenges. Accordingly, early identification and appropriate management of individuals at risk are critical (2). Cervical insufficiency (CI), characterized by painless cervical dilation during the second trimester (3), is a well-recognized contributor to preterm birth (4). Without intervention, most patients with CI deliver within two to three weeks of diagnosis (5), often resulting in pregnancy loss or extremely preterm birth. Furthermore, affected patients face an increased risk for recurrent pregnancy losses or preterm birth in subsequent pregnancies. Despite its clinical significance, the etiology of CI, as well as the underlying mechanisms and predictive markers leading to preterm birth, remain poorly understood.

Cervical cerclage has been performed for the management of CI to reduce perinatal mortality and the incidence of recurrent preterm birth (6), and its use is endorsed by both the American College of Obstetricians and Gynecologists and the Royal College of Obstetricians and Gynaecologists in the United Kingdom (7). While cerclage provides mechanical reinforcement to a structurally compromised cervix, it may also enhance the barrier function of the cervical mucus plug against ascending intrauterine infection (8, 9). Evidence suggests that cervical cerclage can reduce the risk of preterm birth in high-risk women and may lower perinatal mortality (10). However, the precise mechanisms by which cerclage modulates the immunologic microenvironment remain unclear, and the predictive value of the immunologic markers for clinical outcomes has seldom been investigated. A previous study demonstrated that women with CI exhibit increased concentrations of proinflammatory cytokines in cervicovaginal fluid compared with gestational age–matched controls, while systemic cytokine levels often remained unchanged, highlighting a predominantly local immune disturbance at the cervix (11). Cerclage placement was associated with a subsequent decline in local proinflammatory cytokines, suggesting that the procedure may modulate the cervical immune microenvironment beyond providing mechanical support. These observations support the concept that cervical cerclage influences both local barrier function and immune regulation (11–13). Therefore, it is essential to investigate the impact of cervical cerclage on the immunologic markers such as immune-checkpoint regulators (ICRs) and inflammatory cytokines and chemokines (ICKs), which are strongly implicated in the immunopathology of preterm birth in patients with CI. Notably, endocervical samples may serve as reliable indicators of disease status and pregnancy outcomes. Analysis of immunologic factors within these samples may provide novel insight into the underlying mechanisms of CI and highlight their potential utility in predicting preterm birth.

## 2 Materials and methods

### 2.1 Participants and samples

Patients diagnosed with CI who presented to the High-Risk Maternal Neonatal Intensive Care Center at the Kangnam Sacred Heart Hospital, Seoul, Korea, were prospectively enrolled between September 2022 and October 2023. Endocervical swab specimens were collected both before and after emergency cervical cerclage. The samples were collected in the operating room immediately prior to cerclage and subsequently on the day before discharge. The study protocol was approved by the Institutional Review Board of the Kangnam Sacred Heart Hospital (IRB No. HKS 2022-06-010), and written informed consent was obtained from all participants.

Inclusion criteria were: cervical length < 25 mm, internal cervical os dilatation ≥1 cm, cervical effacement of ≥ 50%, and visible fetal membranes at or beyond the external cervical os. Exclusion criteria included maternal age under 18 years, multifetal gestations, fetuses with major structural anomalies, ruptured membranes, active vaginal bleeding, clinical chorioamnionitis, or persistent regular uterine contractions.

Clinical information, including detailed obstetric and gynecologic history, was obtained from electronic medical records. None of the women in this cohort had a documented history of cervical conization or LEEP due to HPV-related cervical intraepithelial neoplasia. Accordingly, prior excisional cervical surgery was not included as a covariate in the multivariable analyses.

Emergency cervical cerclage was performed using a uniconcave balloon device. In cases of bulging fetal membranes, the cervix was gently retracted using two atraumatic forceps, and the inflated balloon was utilized to reposition the membranes into the uterine cavity. Cervical cerclage was then placed using the McDonald technique, with 5 mm polyester tape positioned as high as possible around the cervix. Following balloon deflation, a purse-string suture was secured, and the device was withdrawn from the cervical canal. All procedures were performed by a Maternal-Fetal Medicine specialist using a standardized surgical technique.

### 2.2 Measurement of ICRs and ICKs

Sterile Dacron swabs (Puritan Medical Products, Guilford, ME, USA) were used to obtain endocervical samples before and after cerclage, as the endocervix presents a critical site for assessing the risk of preterm birth. Soluble ICRs were assayed using the MILLIPLEX Human Immuno-Oncology Checkpoint Protein Premixed 17-plex Panel (Merck, Darmstadt, Germany). The measured seventeen ICRs were: soluble B- and T-lymphocyte attenuator (BTLA), cluster of differentiation (CD) 27, CD28, CD40, CD80 (B7-1), CD86 (B7-2), cytotoxic T-lymphocyte-associated protein 4 (CTLA-4), glucocorticoid-induced TNFR-related protein (GITR), ligand for receptor TNFRSF18/AITR/GITR (GITRL), herpesvirus entry mediator (HVEM), inducible T-cell costimulatory (ICOS), lymphocyte-activation gene 3 (LAG-3), programmed cell death protein 1 (PD-1), programmed death-ligand 1 (PD-L1), programmed death-ligand 2 (PD-L2), T-cell immunoglobulin and mucin-domain containing-3 (TIM-3), and toll like receptor 2 (TLR-2).

ICKs were measured using the Human XL Cytokine Luminex Performance Panel Premixed Kit (R&D Systems, Minneapolis, MN, USA). The following 17 cytokines and chemokines were quantified: chemokine (C-C motif) ligand 2 [CCL27 (MCP-1)], CCL3 (MIP-1α), CCL4 (MIP-1β), C-X-C motif chemokine ligand [CXCL10 (IP-10)], granulocyte-macrophage colony-stimulating factor (GM-CSF), interferon (IFN)-α2, IFN-γ, interleukin (IL)-1α, IL-1β, IL-4, IL-6, IL-8, IL-10, IL-12p70, IL-13, IL-17A, and tumor necrosis factor-α (TNF-α). Multiplex assays were conducted according to the manufacturer’s instructions using Luminex-based technology on a Bio-Plex 200 system (Bio-Rad, Hercules, CA, USA).

In brief, each plate included a full set of calibration standards provided by the manufacturer, spanning seven serial dilutions, which were fitted using a five-parameter logistic curve to generate standard curves and calculate analyte concentrations. In addition, high- and low-concentration quality-control samples and internal controls were run on every plate. Inter- and intra-assay coefficients of variation for these controls remained within the ranges recommended by the manufacturers. For low-abundance analytes, values below the lower limit of detection were handled conservatively by assigning half the lower limit concentration for statistical analyses (14, 15). The optimal dilution factor was determined through preliminary measurements performed prior to the main experiment.

All endocervical samples were collected using the same type of sterile swab by Maternal–Fetal Medicine specialists following a standardized protocol. The swab was gently introduced into the endocervical canal, rotated to sample the endocervical mucosa, and withdrawn while avoiding contact with the vaginal wall to minimize contamination from the ectocervix or vagina. Uniform conditions and identical handling and procedures were followed. Immediately after collection, each swab was placed into 1 mL of sample buffer consisting of 1% bovine serum albumin in Tris buffer with 5 mmol/L ethylenediaminetetraacetic acid, 5 mmol/L phenylmethylsulfonyl fluoride, and 0.5 trypsin inhibitor units of aprotinin. After vortexing for 5 seconds at room temperature, the tubes were centrifuged at 1,500 × g for 15 minutes, and the supernatants were frozen at −70 °C until analysis. To further reduce the impact of variability in sampling efficiency and biomass, our primary analyses were based on within-subject paired comparisons using non-parametric paired tests and multivariate methods, which are relatively robust to inter-individual differences.

Clinical data, including demographics, disease state, and pregnancy outcomes, along with quantified ICRs and ICKs profiles, have been deposited in the Figshare repository and are accessible at https://doi.org/10.6084/m9.figshare.30280570.v1.

### 2.3 Statistical analysis

Principal component analysis (PCA) was employed to reduce data dimensionality by transforming the original observed variables into a smaller set of principal components that captured most of the variance. This approach was chosen to address potential multicollinearity among immunologic markers and to facilitate interpretation of the high-dimensional dataset. Prior to PCA, data preprocessing was performed using the pcaMethods package in R (R Foundation for Statistical Computing, Vienna, Austria), with row-wise scaling applied (16). Unit variance scaling was implemented by dividing each value by its respective standard deviation. Multivariate analysis of covariance (MANCOVA) was then conducted with principal component scores (PC1, PC2) as dependent variables to evaluate the effects of cerclage and preterm birth. MANCOVA was selected as it allows simultaneous testing of multiple dependent variables while adjusting for covariates, thereby providing a robust assessment of whether group centroids differ in multivariate space. This approach enabled testing for mean vector differences according to cerclage status and preterm birth while accounting for potential confounders.

Descriptive statistical analyses were performed to compare clinical and immunological variables across groups. Categorical variables were analyzed using the Chi-square test, while paired within-subject comparisons were evaluated with the Wilcoxon signed-rank test. For comparisons between two independent groups, the Mann–Whitney U test was applied. Given the nonparametric distribution of several variables and the presence of multiple testing, nonparametric analyses for relative effects were conducted using the moonBook and nparcomp packages in R, with appropriate *P*-value adjustments to control for type I error.

Multivariate logistic regression analysis was subsequently performed, incorporating variables that demonstrated statistical significance in univariate analyses as covariates. Receiver operating characteristic (ROC) curve analysis was applied to evaluate the predictive performance of combined factors with P < 0.05 in univariate analyses. The discriminatory ability of combined markers was quantified using the area under the ROC curve (AUC), interpreted as follows: acceptable (AUC = 0.7 - 0.8), excellent (AUC = 0.8 - 0.9), and outstanding (AUC > 0.9). In addition, the correlations between ICRs and ICKs were evaluated using Spearman’s rank correlation coefficients, with a focus on markers associated with preterm birth, as determined by the Mann–Whitney U test.

All statistical analyses were performed using ClustVis, a web-based tool for clustering and visualization of multivariate data (University of Tartu, Tartu, Estonia) (17), MedCalc software version 19.8 (MedCalc Software Ltd., Ostend, Belgium), and the moonBook package in R (http://web-r.org/).

## 3 Results

### 3.1 Characteristics of participants and samples

A total of 128 endocervical swab samples were prospectively collected from 64 patients diagnosed with CI who underwent cervical cerclage at the High-Risk Maternal Neonatal Intensive Care Center of the Kangnam Sacred Heart Hospital, Seoul, Korea. Samples were obtained before and after cerclage placement (n = 64 each) (**Supplementary Table 1**). The study cohort comprised 17 patients who underwent emergent cerclage and 47 who received urgent cerclage. The median gestational age at the time of cerclage placement was 22.0 weeks [interquartile range between 1^st^ and 3^rd^ quartile (IQR): 21.0–23.8 weeks], and the median duration of hospitalization was 4.5 days (IQR: 3.0–7.6 days). The median interval between pre- and post-cerclage was 4.0 days (IQR: 3.0–7.0 days). On admission, bulging membranes were found in 17 patients (26.6%). Of the 32 participants with complete clinical outcome data (50.0% of the cohort), 13 (40.6%) experienced preterm birth. Detailed clinical outcomes are presented in **Supplementary Table 1**.

### 3.2 Effect of cervical cerclage on ICRs and ICKs

**Figure 1:**
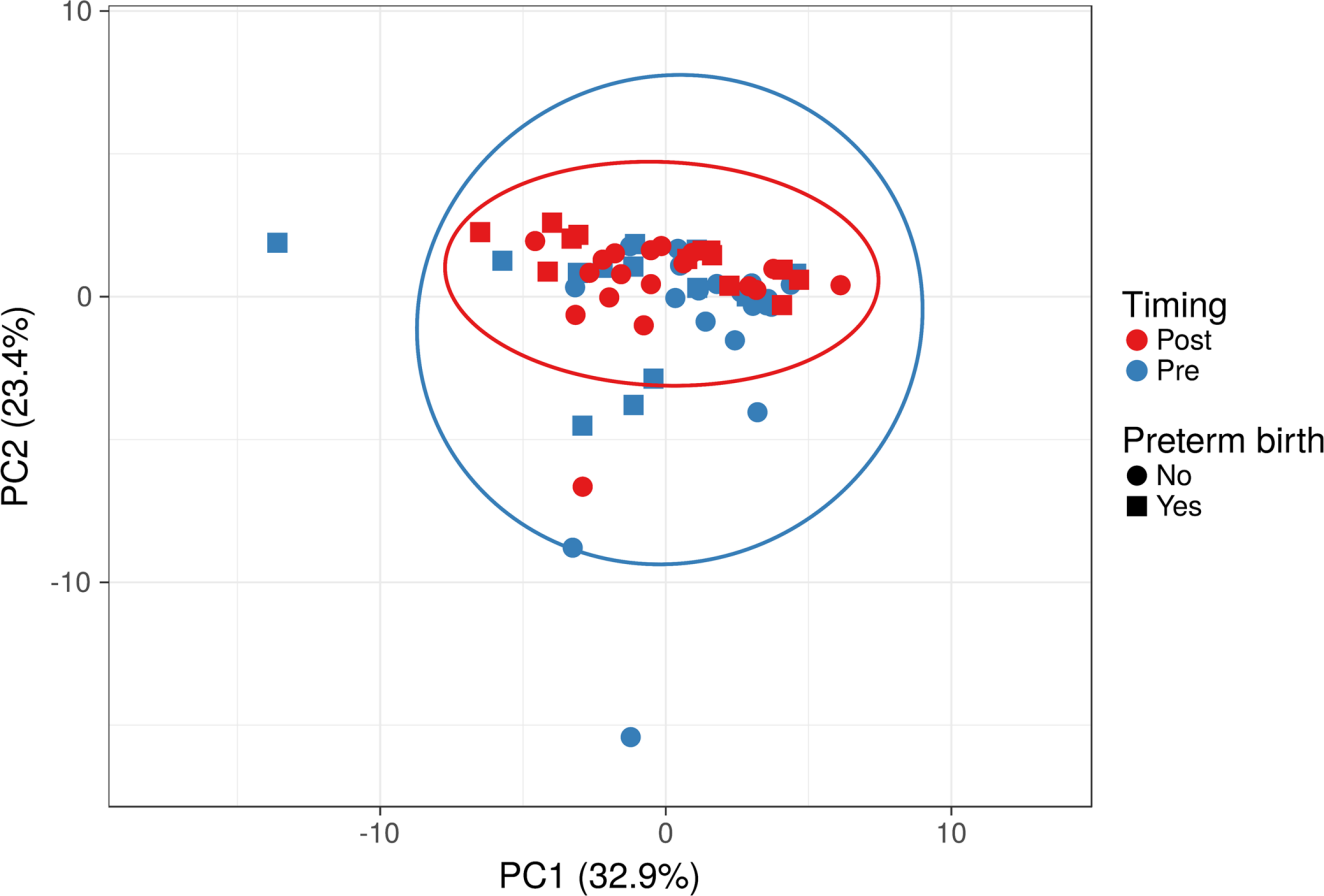
Principal component analysis plots for soluble immune-checkpoint regulators and inflammatory cytokines and chemokines according to sampling time and preterm birth status. Prediction ellipses, illustrated as red and blue lines, represent a 0.95 probability.

To evaluate the overall effects of cervical cerclage on soluble ICRs and ICKs, PCA was conducted, incorporating preterm birth as a clinical outcome (**Figure 1**). The PCA plot for endocervical samples displays principal component 1 (PC1) and principal component 2 (PC2) on the X and Y axes, accounting for 32.9% and 23.4% of the total variance, respectively (**Figure 1**). Pre-cerclage samples exhibited greater variability compared to post-cerclage samples. The PCA scores, loading values, and explained variance for soluble ICRs and ICKs are presented in **Supplementary Tables 2**–**S4**, respectively.

Principal component analysis identified two major components (PC1 and PC2), which were subsequently analyzed using MANCOVA with cervical cerclage (pre- vs. post-cerclage) and preterm birth status as independent variables. The overall multivariate test revealed borderline group differences for both cerclage status and preterm birth (*P* = 0.058 and *P* = 0.063, respectively), while the interaction between the two factors was not significant. In *post-hoc* univariate analyses, PC1 was primarily associated with preterm birth (*P* = 0.084), whereas PC2 was significantly associated with changes pre- and post-cerclage (*P* = 0.021). Group centroids and separation along PC1 and PC2 are illustrated in **Supplementary Figure 1**.

### 3.3 ICRs and ICKs changes before and after cervical cerclage

**Figure 2:**
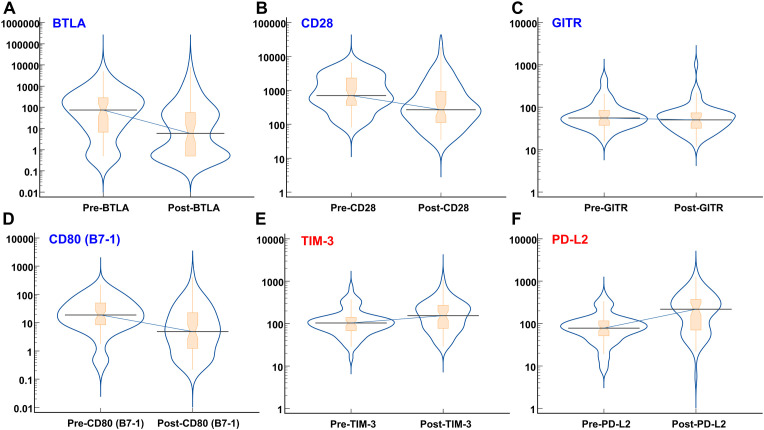
Comparison of immune-checkpoint regulators between pre- and post-cerclage samples in patients with cervical insufficiency, analyzed using the paired Wilcoxon-rank test. **(A)** BTLA; **(B)** CD28; **(C)** GITR; **(D)** CD80 (B7-1); **(E)** TIM-3; and **(F)** PD-L2. Increased variables are shown in red, whereas decreased variables are shown in blue. The horizontal line within each box represents the median of the data.

**Figure 3:**
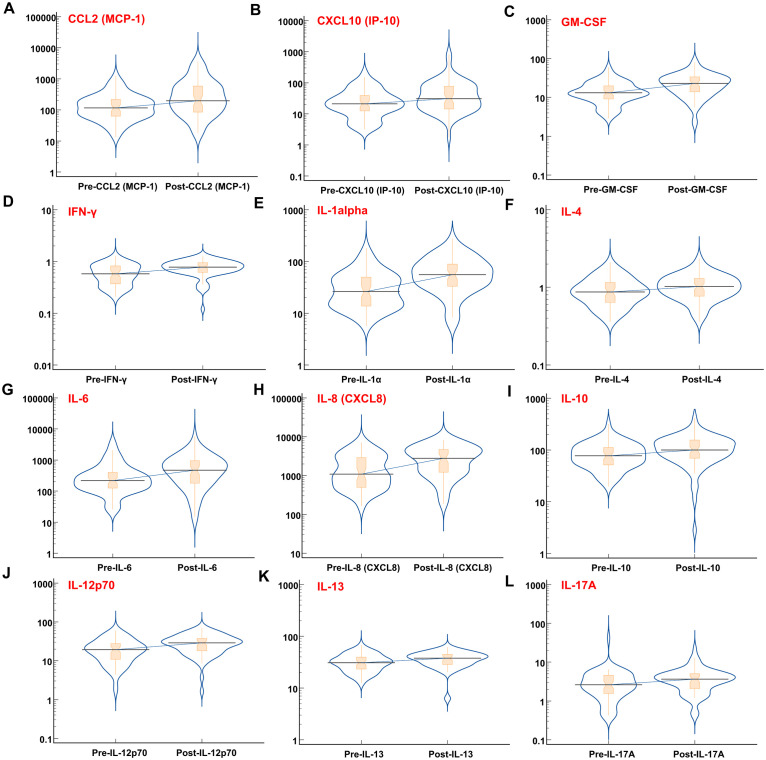
Comparison of inflammatory cytokines and chemokines between pre- and post-cerclage samples in patients with cervical insufficiency, analyzed using the paired Wilcoxon-rank test. **(A)** CCL2 (MCP-1); **(B)** CXCL10 (IP-10); **(C)** GM-CSF; **(D)** IFN-γ; **(E)** IL-1α; **(F)** IL-4; **(G)** IL-6; **(H)** IL-8 (CXCL8); **(I)** IL-10; **(J)** IL-12p70; **(K)** IL-13; and **(L)** IL-17A. Increased cytokines are shown in red. The horizontal line within each box represents the median of the data.

Pairwise comparisons of soluble ICR and ICK levels in pre- and post-cervical cerclage samples were performed in 64 patients with CI (**Table 1**). Among the ICRs, the soluble BTLA, CD28, GITR, and CD80 (B7-1) levels were significantly decreased in post-cerclage samples. In contrast, soluble TIM-3 and PD-L2 levels were significantly higher in post-cerclage samples than pre-cerclage samples (**Figure 2**). For the ICKs, 12 of 17 cytokines and chemokines, including CCL2 (MCP-1), CXCL10 (IP-10), GM-CSF, IFN-γ, IL-1α, IL-4, IL-6, IL-8 (CXCL8), IL-10, IL-12p70, IL-13, and IL-17A, showed significant increases in post-cerclage samples (**Figure 3**).

**Table 1:** Comparison of soluble immune-checkpoint regulators and inflammatory cytokines and chemokines in patients with cervical insufficiency before and after cervical cerclage.

Variables^a^		Paired swab samples		
		Before cerclage	After cerclage	P-value
ICRs, pg/ml	BTLA	74.2 (6.7–281.6)	5.9 (0.5–56.8)	0.002
	CD27	22.3 (4.8–59.0)	15.3 (4.8–55.8)	0.573
	CD28	700.5 (363.3–2319.8)	267.0 (113.0–927.7)	0.002
	TIM-3	102.8 (68.2–139.6)	153.9 (77.4–270.0)	< 0.001
	HVEM	287.2 (210.9–561.5)	415.6 (242.7–623.6)	0.113
	CD40	64.1 (47.8–144.9)	82.8 (52.1–166.2)	0.082
	GITR	55.9 (37.4–84.1)	50.6 (32.0–73.7)	0.021
	LAG-3	275.9 (75.4–952.3)	230.2 (82.7–727.7)	0.354
	TLR-2	390.1 (210.5–619.0)	430.8 (227.4–640.7)	0.753
	GITRL	0.5 (0.5–22.5)	0.5 (0.5–9.9)	0.573
	PD-1	171.2 (42.6–205.5)	163.0 (36.3–194.4)	0.918
	CTLA-4	4.4 (3.6–6.6)	4.3 (2.7–5.9)	0.352
	CD80 (B7-1)	18.6 (8.4–49.6)	4.9 (1.2–22.5)	0.002
	CD86 (B7-2)	9.8 (3.9–33.0)	8.4 (1.9–28.6)	0.516
	PD-L1	12.0 (5.3–31.2)	6.7 (4.4–16.7)	0.147
	PD-L2	77.8 (51.5–115.4)	218.1 (70.4–370.1)	< 0.001
	ICOS	46.5 (5.5–153.6)	58.7 (16.3–132.5)	0.766
ICKs, pg/ml	CCL2 (MCP-1)	116.4 (63.1–218.3)	197.0 (85.4–584.9)	0.002
	CCL3 (MIP-1α)	56.6 (28.0–119.2)	75.0 (39.8–145.0)	0.226
	CCL4 (MIP-1β)	216.0 (156.5–424.4)	277.2 (188.5–541.7)	0.279
	CXCL10 (IP-10)	20.9 (12.4–38.9)	31.0 (14.4–77.3)	< 0.001
	GM-CSF	13.1 (9.1–19.8)	22.8 (14.0–33.8)	< 0.001
	IFN-α2	6.0 (4.5–7.5)	6.4 (4.8–8.3)	0.596
	IFN-γ	0.6 (0.4–0.8)	0.8 (0.6–0.9)	< 0.001
	IL-1α	26.2 (13.8–49.9)	55.4 (33.3–89.1)	< 0.001
	IL-1β	142.3 (61.2–535.2)	275.9 (112.1–551.2)	0.204
	IL-4	0.9 (0.6–1.2)	1.0 (0.8–1.3)	0.011
	IL-6	216.5 (125.1–403.2)	466.9 (180.0–951.8)	0.001
	IL-8 (CXCL8)	1086.6 (497.8–2898.9)	2784.9 (1219.6–4694.7)	0.001
	IL-10	77.4 (51.5–111.5)	99.4 (68.5–153.4)	0.003
	IL-12p70	19.5 (10.9–27.7)	29.0 (18.1–37.5)	< 0.001
	IL-13	30.8 (23.3–39.5)	37.6 (28.5–44.8)	0.006
	IL-17A	2.6 (1.6–4.6)	3.7 (2.1–5.0)	0.008
	TNF-α	13.1 (6.4–19.2)	16.5 (9.0–32.9)	0.094

^a^ Values are presented as median (1st quartile-3rd quartile).

ICRs, immune-checkpoint regulators; ICKs, inflammatory cytokines and chemokines.

### 3.4 ICRs and ICKs in patients with bulging membrane

Bulging membranes represent a severe clinical condition in which the fetal membranes protrude through a dilated cervical os due to cervical weakness. This situation typically necessitates emergent cerclage and is frequently associated with adverse outcomes, including preterm birth. All patients with bulging membranes (n = 17) underwent emergent cerclage (**Table 2**). The median duration of hospitalization was significantly longer in these patients (9.0 days vs. 4.0 days, *P* < 0.001). Gestational age at cerclage was earlier in the membrane bulging group compared with the non-bulging group (21.0 vs. 22.0 weeks). Notably, four patients (23.5%) underwent two cerclage procedures, having previously received cerclage earlier in the pregnancy.

**Table 2:** Clinical characteristics of patients with cervical insufficiency.

Variables^a^	Preterm birth			Membrane bulging		
	No	Yes	P-value	No	Yes	P-value
No Age, year	19 34.0 (31.0–36.0)	13 33.0 (33.0–36.0)	- 0.714	47 34.0 (30.0–37.0)	17 33.0 (31.0–37.0)	- 0.921
Hospitalization, day	5.0 (3.5– 6.0)	7.0 (4.0–19.0)	0.088	4.0 (3.0– 5.0)	9.0 (8.0–15.0)	< 0.001
Operation			0.140			< 0.001
- Emergent Cerclage	3 (15.8%)	6 (46.2%)		0 (0.0%)	17 (100.0%)	
- Urgent cerclage	16 (84.2%)	7 (53.8%)		47 (100.0%)	0 (0.0%)	
Membrane bulging			0.058			
- No	16 (84.2%)	6 (46.2%)				
- Yes	3 (15.8%)	7 (53.8%)				
Gestational age at cerclage, week	23.0 (21.8–26.8)	21.0 (18.0–23.0)	0.014	22.0 (21.0–24.2)	21.0 (17.0–23.0)	0.023
Number of cerclages			1.000			0.022
- One	17 (89.5%)	11 (84.6%)		46 (97.9%)	13 (76.5%)	
- Two	2 (10.5%)	2 (15.4%)		1 (2.1%)	4 (23.5%)	
Prior preterm birth			0.375			0.929
- No	16 (84.2%)	13 (100.0%)		41 (87.2%)	14 (82.4%)	
- Yes	3 (15.8%)	0 (0.0%)		6 (12.8%)	3 (17.6%)	

^a^ Values are presented as median (1st quartile-3rd quartile) or number (%).

When ICRs were measured in patients with CI before cerclage, levels of TIM-3, HVEM, and CD40 were higher in the membrane bulging group compared to the non-membrane bulging group (**Table 3**). For ICKs sampled before cerclage, all of the cytokines and chemokines showed significant increases, with the exception of IL-8 (CXCL8), which was decreased. Most ICRs in the post-cerclage samples, including BTLA, CD28, GITR, GITRL, PD-1, CTLA-4, CD80 (B7-1), CD86 (B7-2), and PD-L1, were decreased in the membrane bulging group compared to the non-membrane bulging group (**Table 4**). Among ICKs in the post-cerclage samples, only IL-1β showed a reduced level in the membrane bulging group.

**Table 3:** Comparison of soluble immune-checkpoint regulators and inflammatory cytokines and chemokines in patients with cervical insufficiency before cerclage.

Variables^a^		Preterm birth			Membrane bulging		
		No	Yes	P-value	No	Yes	P-value
ICRs, pg/ml	bPre-BTLA	199.8 (5.0–283.2)	98.8 (17.1–288.3)	0.908	90.8 (10.7–312.0)	58.1 (5.0–113.8)	0.234
	Pre-CD27	28.5 (2.7–53.7)	65.3 (11.4–83.6)	0.537	15.5 (4.1–54.5)	28.5 (8.4–75.2)	0.414
	Pre-CD28	1165.7 (488.8–2833.9)	687.8 (509.2–1957.8)	0.715	687.8 (339.1–2319.8)	713.1 (380.3–1957.8)	0.558
	Pre-TIM-3	101.3 (78.4–129.4)	121.2 (87.0–151.7)	0.448	91.5 (62.1–126.9)	122.2 (90.2–217.5)	0.039
	Pre-HVEM	268.8 (241.6–472.1)	443.6 (357.5–778.9)	0.136	261.5 (212.0–436.8)	545.0 (210.7–1046.9)	0.044
	Pre-CD40	64.8 (56.9–82.7)	80.0 (44.1–183.9)	0.677	55.1 (39.0–85.6)	134.6 (80.0–214.9)	0.001
	Pre-GITR	55.7 (46.6–74.4)	66.7 (49.3–78.8)	0.466	59.6 (43.5–86.5)	47.1 (32.6–66.7)	0.123
	Pre-LAG-3	383.8 (224.4–1318.8)	253.1 (91.6–699.8)	0.140	275.9 (87.1–1088.1)	138.1 (68.2–600.8)	0.323
	Pre-TLR-2	394.5 (297.0–584.8)	601.9 (401.0–706.9)	0.126	362.9 (225.1–595.5)	401.0 (191.3–696.9)	0.670
	Pre-GITRL	13.2 (0.5–24.1)	11.4 (0.5–26.7)	0.936	0.5 (0.5–23.4)	0.5 (0.5–21.9)	0.826
	Pre-PD-1	174.0 (42.6–211.1)	170.3 (51.2–187.0)	0.527	174.9 (76.4–214.3)	150.0 (32.9–171.2)	0.063
	Pre-CTLA-4	4.7 (3.7– 6.1)	4.6 (4.2–13.6)	0.388	4.5 (3.7– 6.2)	4.2 (3.0– 6.9)	0.287
	Pre-CD80 (B7-1)	25.4 (12.5–46.6)	34.1 (10.7–53.4)	0.878	18.6 (7.6–53.4)	18.7 (9.9–44.5)	0.779
	Pre-CD86 (B7-2)	7.9 (4.3–42.3)	16.3 (9.8–69.0)	0.701	9.8 (4.3–32.2)	10.7 (1.3–30.4)	0.939
	Pre-PD-L1	13.6 (8.7–24.0)	19.6 (7.2–41.9)	0.734	12.0 (5.3–27.8)	11.9 (5.8–33.9)	0.600
	Pre-PD-L2	72.3 (51.5–78.6)	101.9 (63.7–121.5)	0.125	76.9 (45.5–113.6)	93.2 (63.7–121.5)	0.323
	Pre-ICOS	124.8 (33.3–177.2)	46.8 (19.8–178.1)	0.591	33.2 (5.5–137.3)	71.1 (19.8–178.1)	0.478
ICKs, pg/ml	Pre-CCL2 (MCP-1)	99.3 (59.3–194.2)	269.4 (147.4–413.6)	0.003	84.8 (59.4–151.3)	269.4 (201.8–633.4)	< 0.001
	Pre-CCL3 (MIP-1α)	57.6 (33.6–85.3)	87.3 (47.5–183.8)	0.170	50.0 (26.4–77.9)	141.7 (87.3–200.0)	< 0.001
	Pre-CCL4 (MIP-1β)	208.7 (158.6–315.5)	311.4 (182.3–796.4)	0.147	190.2 (135.8–301.7)	458.0 (237.2–835.2)	0.001
	Pre-CXCL10 (IP-10)	20.6 (14.1–33.1)	32.3 (20.9–97.0)	0.033	15.6 (7.5–29.3)	35.5 (24.5–96.3)	0.001
	Pre-GM-CSF	12.8 (7.4–16.7)	22.4 (13.8–26.9)	0.006	11.1 (7.3–14.0)	25.3 (19.2–31.3)	< 0.001
	Pre-IFN-α2	5.3 (4.7– 6.9)	6.4 (4.9– 9.4)	0.120	5.3 (4.2– 7.2)	7.3 (6.2– 9.4)	0.001
	Pre-IFN-γ	0.5 (0.3– 0.7)	0.8 (0.6– 0.9)	0.055	0.5 (0.3– 0.6)	0.9 (0.7– 0.9)	< 0.001
	Pre-IL-1α	25.9 (13.2–41.5)	54.8 (33.1–82.1)	0.042	19.8 (12.9–38.8)	56.1 (38.4–82.1)	< 0.001
	Pre-IL-1β	133.2 (74.1–291.3)	271.1 (120.8–1149.4)	0.108	98.1 (52.4–276.0)	271.1 (197.6–834.1)	0.005
	Pre-IL-4	0.9 (0.6– 1.2)	1.0 (0.8– 1.2)	0.134	0.8 (0.6– 1.0)	1.0 (0.8– 1.4)	0.006
	Pre-IL-6	186.8 (109.4–332.1)	402.8 (287.5–1007.9)	0.006	167.8 (101.9–290.5)	477.9 (285.8–1361.3)	< 0.001
	Pre-IL-8 (CXCL8)	1095.6 (702.5–2240.6)	2593.7 (1471.3–4751.5)	0.065	872.9 (435.1–1525.3)	3226.4 (2427.0–3822.1)	< 0.001
	Pre-IL-10	56.5 (47.4–101.7)	107.8 (74.9–143.8)	0.042	62.1 (49.1–90.5)	133.4 (107.8–175.2)	< 0.001
	Pre-IL-12p70	16.8 (10.9–25.1)	26.3 (20.8–40.6)	0.055	15.4 (8.6–23.8)	31.7 (22.0–43.8)	< 0.001
	Pre-IL-13	28.5 (20.3–38.3)	36.0 (30.8–44.8)	0.134	30.7 (20.3–36.0)	36.9 (30.8–50.5)	0.001
	Pre-IL-17A	2.1 (1.4– 3.1)	4.7 (3.0– 6.2)	0.005	2.2 (1.2– 3.1)	5.7 (3.0– 6.2)	< 0.001
	Pre-TNF-α	13.5 (6.4–17.9)	18.0 (15.3–45.7)	0.063	8.8 (5.8–16.8)	24.5 (15.3–45.3)	< 0.001

^a^ Values are presented as median (1st quartile-3rd quartile); ^b^Pre-, pre-cerclage.

ICRs, immune-checkpoint regulators; ICKs, inflammatory cytokines and chemokines.

**Table 4:** Comparison of soluble immune-checkpoint regulators and inflammatory cytokines and chemokines in patients with cervical insufficiency after cerclage.

Variables^a^		Preterm birth			Membrane bulging		
		No	Yes	P-value	No	Yes	P-value
ICRs, pg/ml	bPost-BTLA	6.7 (0.5–50.7)	0.5 (0.5–27.0)	0.389	23.8 (0.5–58.1)	0.5 (0.5– 5.0)	0.013
	Post-CD27	10.7 (2.7–36.2)	11.9 (0.5–25.0)	1.000	15.5 (7.2–57.0)	11.4 (0.5–29.2)	0.289
	Post-CD28	341.4 (180.3–839.4)	170.6 (81.4–244.1)	0.074	402.8 (220.6–1153.2)	103.4 (62.4–197.1)	< 0.001
	Post-TIM-3	142.9 (91.1–246.9)	156.5 (75.4–209.3)	0.623	151.3 (73.4–270.0)	156.5 (84.9–253.9)	0.868
	Post-HVEM	416.4 (223.7–533.7)	323.7 (295.9–675.8)	0.940	421.7 (249.5–611.9)	353.8 (203.6–979.9)	0.976
	Post-CD40	74.0 (41.9–136.4)	107.8 (58.8–143.3)	0.677	81.0 (56.2–162.7)	101.5 (51.1–211.4)	0.775
	Post-GITR	46.0 (37.7–65.8)	39.6 (26.4–46.0)	0.234	57.6 (39.0–78.8)	36.0 (19.9–55.2)	0.006
	Post-LAG-3	400.8 (151.1–867.0)	96.6 (44.6–218.7)	0.025	270.8 (61.2–1251.5)	110.5 (91.6–191.7)	0.056
	Post-TLR-2	423.5 (226.7–584.4)	389.2 (182.9–578.1)	1.000	450.1 (241.4–629.7)	318.7 (172.9–646.2)	0.403
	Post-GITRL	0.5 (0.5– 9.6)	0.5 (0.5– 0.5)	0.063	2.4 (0.5–16.8)	0.5 (0.5– 0.5)	0.001
	Post-PD-1	160.2 (53.5–206.9)	144.4 (26.1–169.4)	0.139	170.3 (39.8–219.9)	62.6 (26.1–160.2)	0.015
	Post-CTLA-4	4.9 (2.8– 5.7)	3.4 (2.5– 3.7)	0.030	4.9 (3.7– 6.8)	2.6 (2.0– 3.9)	< 0.001
	Post-CD80 (B7-1)	8.7 (1.7–19.3)	1.8 (0.5– 4.0)	0.083	9.0 (2.2–30.8)	1.8 (0.5– 2.8)	0.001
	Post-CD86 (B7-2)	10.1 (4.3–28.1)	6.1 (0.5– 7.9)	0.046	11.6 (4.3–35.5)	2.5 (0.5– 8.0)	0.001
	Post-PD-L1	10.3 (4.4–18.7)	4.8 (3.7– 6.1)	0.046	10.3 (4.4–25.0)	5.8 (3.7– 6.9)	0.040
	Post-PD-L2	201.5 (71.2–341.7)	159.0 (80.8–218.6)	0.762	240.5 (71.3–451.1)	102.7 (71.9–218.6)	0.123
	Post-ICOS	46.1 (19.8–132.5)	58.7 (19.8–72.5)	0.758	58.7 (19.8–137.3)	58.7 (12.8–84.9)	0.364
ICKs, pg/ml	Post-CCL2 (MCP-1)	235.7 (171.3–584.9)	204.1 (158.3–373.1)	0.705	196.0 (79.7–621.7)	204.1 (99.4–373.1)	0.821
	Post-CCL3 (MIP-1α)	78.4 (43.2–124.2)	73.8 (36.4–142.2)	1.000	78.4 (41.3–162.5)	70.5 (37.2–135.7)	0.451
	Post-CCL4 (MIP-1β)	271.6 (149.8–514.5)	235.3 (203.2–615.0)	1.000	282.8 (193.7–504.4)	255.7 (182.3–615.0)	0.659
	Post-CXCL10 (IP-10)	37.7 (21.3–77.3)	30.1 (14.8–49.4)	0.472	28.8 (14.4–75.2)	37.7 (17.3–84.2)	0.438
	Post-GM-CSF	23.5 (17.9–32.7)	17.0 (9.9–26.2)	0.502	22.9 (14.0–33.8)	21.2 (16.0–31.3)	1.000
	Post-IFN-α2	5.9 (5.1– 7.5)	6.2 (4.2– 7.9)	0.848	6.8 (5.2– 8.5)	4.9 (4.2– 6.8)	0.054
	Post-IFN-γ	0.8 (0.6– 0.9)	0.7 (0.5– 0.9)	0.729	0.8 (0.7– 0.9)	0.7 (0.6– 0.9)	0.178
	Post-IL-1α	44.2 (33.9–73.2)	66.9 (27.6–74.1)	0.910	59.7 (37.9–100.4)	44.2 (30.8–70.3)	0.215
	Post-IL-1β	317.9 (102.4–533.9)	139.9 (61.4–659.6)	0.910	328.2 (140.5–572.5)	86.2 (34.9–389.4)	0.007
	Post-IL-4	1.0 (0.8– 1.2)	0.8 (0.7– 1.2)	0.514	1.0 (0.8– 1.3)	1.0 (0.7– 1.3)	0.796
	Post-IL-6	467.7 (217.0–856.5)	309.5 (160.3–645.8)	0.472	553.5 (257.3–959.0)	309.5 (86.6–758.3)	0.137
	Post-IL-8 (CXCL8)	2771.5 (1219.6–5235.7)	1532.6 (714.2–6144.4)	0.880	3548.2 (1558.4–4845.6)	1425.0 (1037.3–3661.3)	0.055
	Post-IL-10	100.5 (74.9–141.5)	89.7 (63.9–129.8)	0.478	100.5 (69.4–151.7)	99.4 (74.9–157.3)	0.867
	Post-IL-12p70	29.0 (19.7–33.7)	28.2 (12.7–34.5)	0.908	30.4 (19.5–37.5)	26.3 (15.4–35.8)	0.394
	Post-IL-13	38.4 (31.7–42.5)	36.9 (27.7–40.6)	0.833	36.9 (29.6–44.8)	38.4 (28.5–42.3)	0.796
	Post-IL-17A	3.3 (2.2– 4.6)	3.6 (1.6– 5.7)	0.564	3.6 (2.9– 4.9)	3.3 (1.6– 5.7)	0.503
	Post-TNF-α	13.8 (7.9–31.9)	13.5 (6.7–29.7)	0.631	18.1 (10.2–34.5)	14.4 (6.7–19.4)	0.128

^a^ Values are presented as median (1st quartile-3rd quartile). ^b^Post-, Post-cerclage.

ICRs, immune-checkpoint regulators; ICKs, inflammatory cytokines and chemokines.

### 3.5 ICRs and ICKs associated with preterm birth

**Figure 4:**
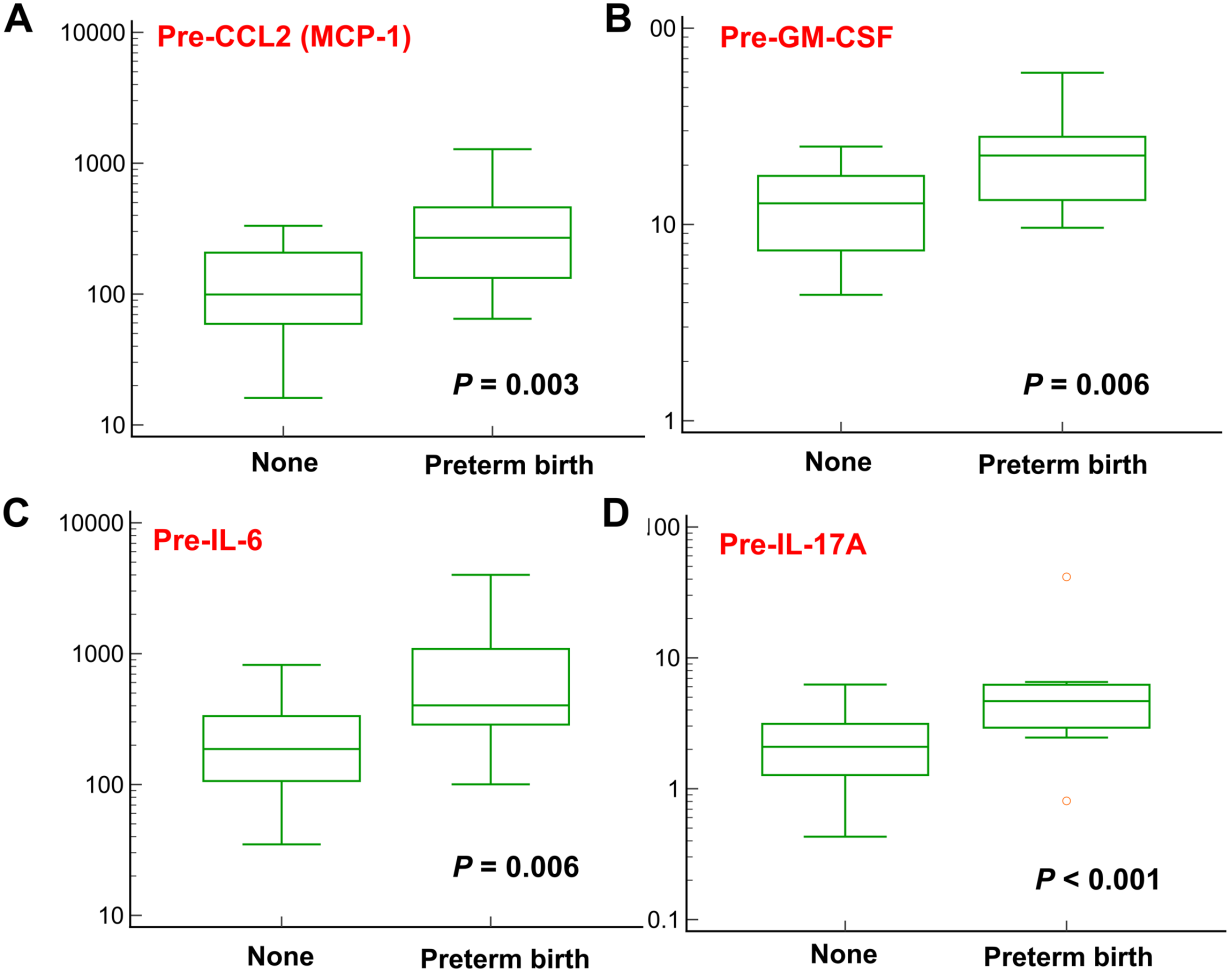
Distribution of inflammatory cytokines and chemokines associated with preterm birth in patients with cervical insufficiency. Plots for **(A)** Pre-CCL2 (MCP-1); **(B)** Pre-GM-CSF; **(C)** Pre-IL-6; and **(D)** Pre-IL-17A. Cytokines with *P*-value < 0.01 are illustrated.

**Figure 5:**
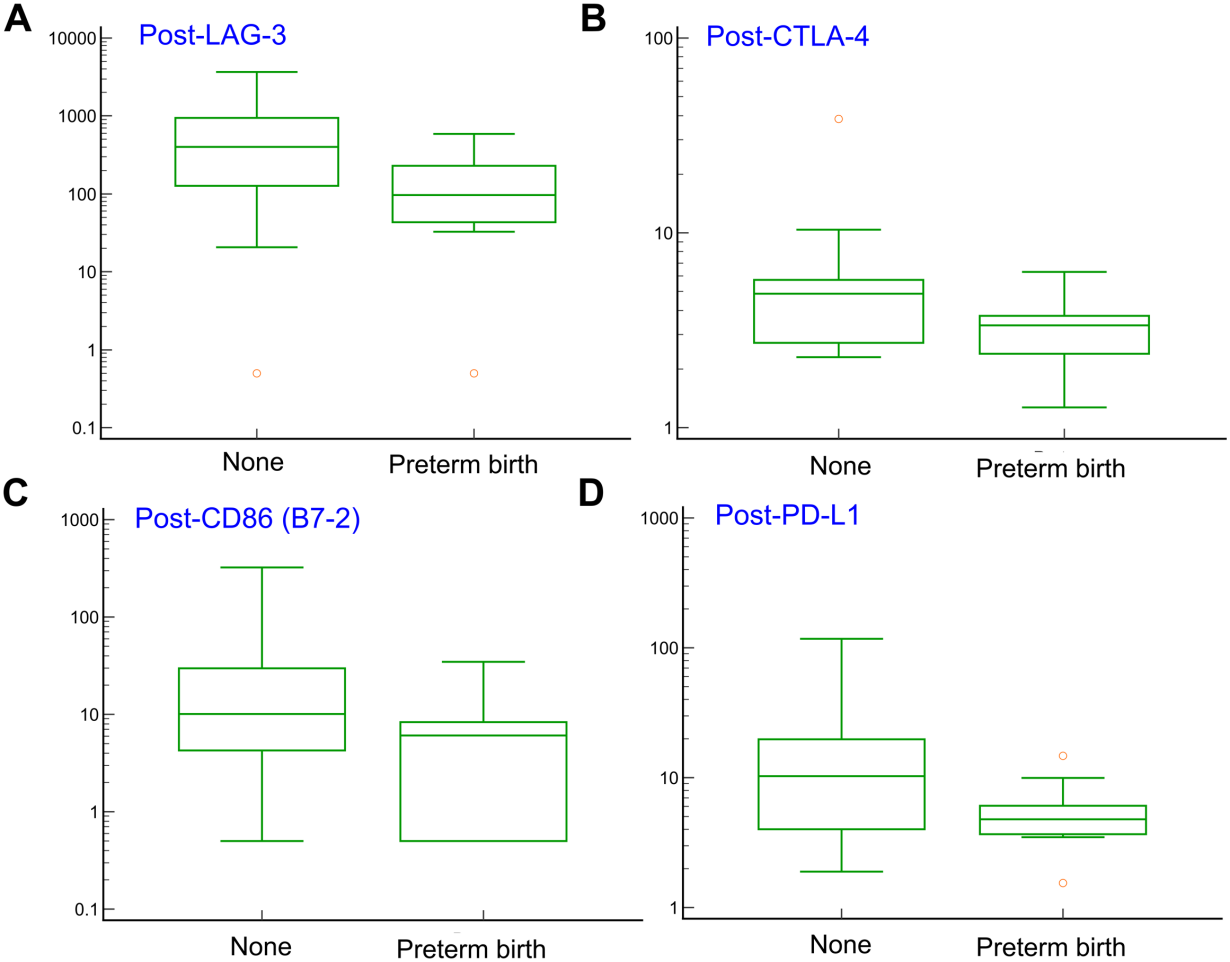
Distribution of immune-checkpoint regulators related to preterm birth cases in patients with cervical insufficiency. Plots for **(A)** Post-LAG-3; **(B)** Post-CTLA-4; **(C)** Post-CD86 (B7-2); and **(D)** Post-PD-L1.

**Figure 6:**
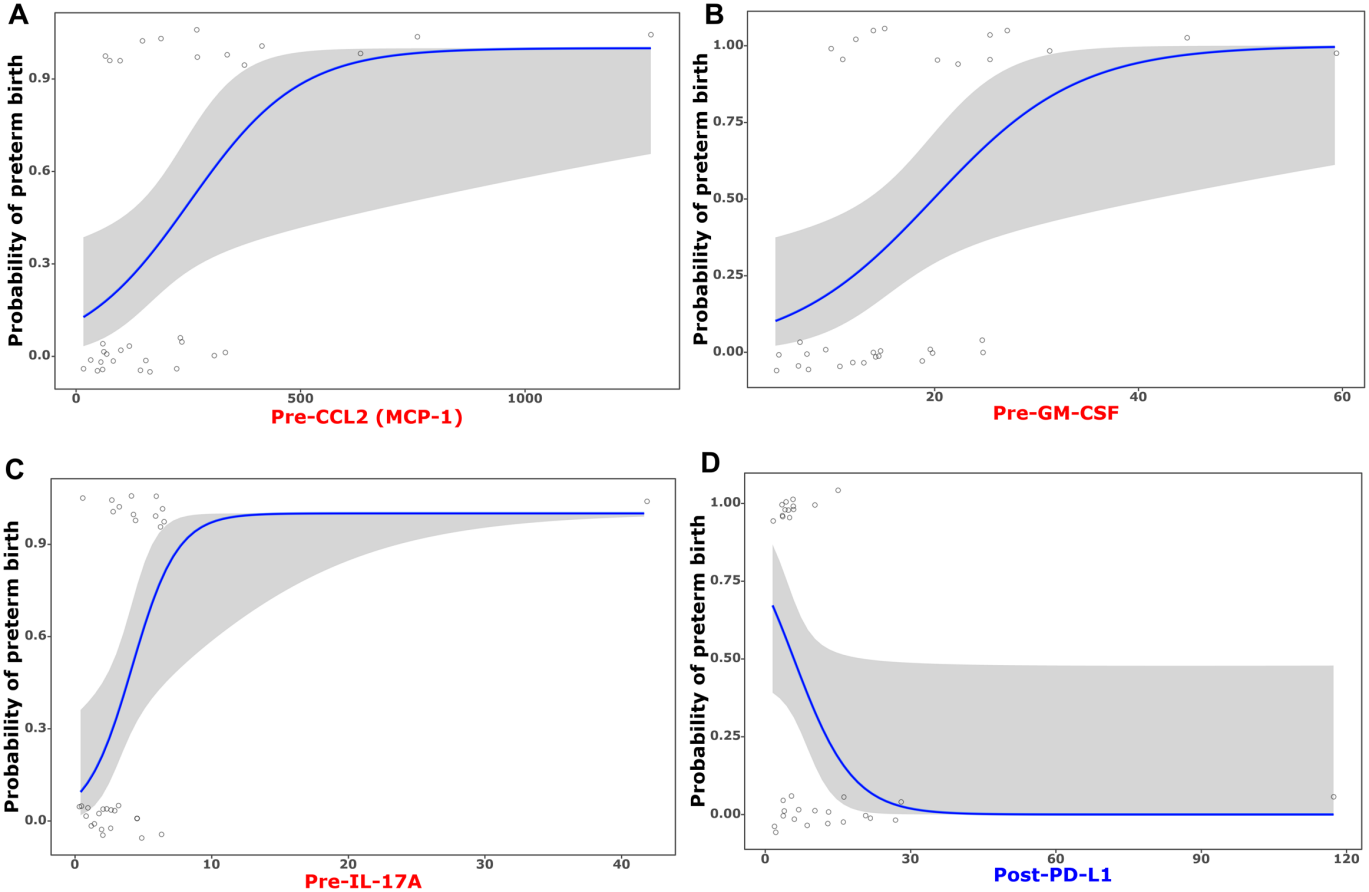
Univariate analysis of immune-checkpoint regulators and inflammatory cytokines and chemokines for predicting preterm birth. Plots show **(A)** Pre-CCL2 (MCP-1); **(B)** Pre-GM-CSF; **(C)** Pre-IL-17A; and **(D)** Post-PD-L1.

Preterm birth, as the major pregnancy outcome, was associated with an earlier gestational age at cerclage and showed a marginally significant association with membrane bulging (**Table 2**). In addition, seven markers measured before cerclage were elevated in the preterm birth group (**Table 3**). Of these, four markers with *P*-values < 0.010, CCL2 (MCP-1), GM-CSF, IL-6, and IL-17A, are shown in **Figure 4**. In contrast, post-cerclage levels of LAG-3, CTLA-4, CD86 (B7-2), and PD-L1 were lower in the preterm birth group (**Table 4**, **Figure 5**). In univariate analysis, pre-cerclage CCL2 (MCP-1), GM-CSF, IL-17A, and post-cerclage PD-L1 were associated with preterm birth (*P* < 0.05). In multivariate analysis, pre-cerclage IL-17A remained independently significant (**Table 5**; **Figure 6**).

**Table 5:** Univariate and multivariate analyses for predicting preterm birth.

Variables	Univariate		Multivariate^a^	
	OR (95% CI)	P-value	OR (95% CI)	P-value
^b^Pre-CCL2 (MCP-1)	1.0 (1.0–1.0)	0.023		
Pre-CXCL10 (IP-10)	1.0 (1.0–1.1)	0.052		
Pre-GM-CSF	1.2 (1.0–1.3)	0.019		
Pre-IL-1α	1.0 (1.0–1.1)	0.065		
Pre-IL-6	1.0 (1.0–1.0)	0.073		
Pre-IL-10	1.0 (1.0–1.0)	0.075		
Pre-IL-17A	1.8 (1.2–3.1)	0.013	2.3 (1.3–5.2)	0.014
^c^Post-LAG-3	1.0 (1.0–1.0)	0.067		
Post-CTLA-4	0.6 (0.3–0.9)	0.052		
Post-CD86 (B7-2)	1.0 (0.9–1.0)	0.145		
Post-PD-L1	0.9 (0.7–1.0)	0.050	0.8 (0.5–0.9)	0.057

^a^ The results of the finally selected model. ^b^Pre-; pre-cerclage, ^c^Post-; Post-cerclage.

OR, odds ratio; CI, confidence interval.

**Figure 7:**
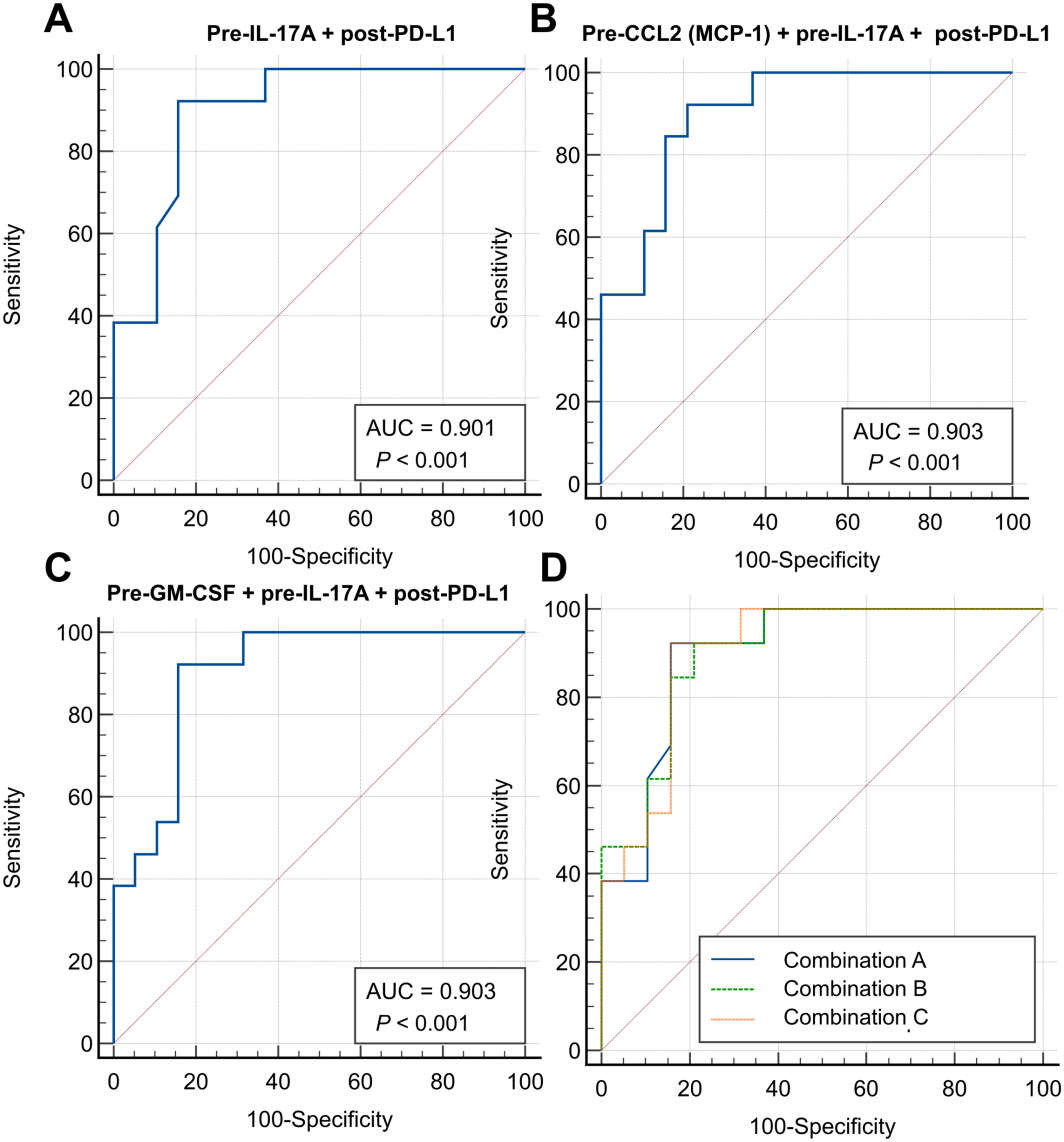
Area under the receiver operating characteristic curves (AUCs) of immune-checkpoint regulators and inflammatory cytokines and chemokines for predicting preterm birth. **(A)** Pre-IL-17A + post-PD-L-1; **(B)** Pre-CCL2 (MCP-1) + pre-IL-17A + post-PD-L1; **(C)** Pre-GM-CSF + pre-IL-17A + post-PD-L1; and **(D)** Comparison of these three AUCs. AUCs with values > 0.9 are illustrated. Combination A = Pre-IL-17A + post-PD-L-1; Combination B = Pre-CCL2 (MCP-1) + pre-IL-17A + post-PD-L1; and Combination C = Pre-GM-CSF + pre-IL-17A + post-PD-L1. There were no significant differences among combinations A, B, and C.

The diagnostic performance of individual and combined predictors was evaluated using AUC analysis for pre-cerclage CCL2 (MCP-1), GM-CSF, and IL-17A, and post-cerclage PD-L1 levels (**Table 6**). Among these, the combination of pre-cerclage IL-17A and post-cerclage PD-L1 yielded an excellent AUC of 0.901. A three-marker combination of pre-cerclage CCL2 (MCP-1), pre-cerclage IL-17A, and post-cerclage PD-L1 demonstrated an AUC of 0.903, which was identical to that of the combination including pre-cerclage GM-CSF, pre-cerclage IL-17A, and post-cerclage PD-L1. There was no statistically significant difference between the AUC of the two-marker model (pre-cerclage IL-17A + post-cerclage PD-L1) and the three-marker models (**Figure 7**).

**Table 6:** Performance of immune-checkpoint regulators and inflammatory cytokines for predicting preterm birth.

Predictors^a^	AUC (95% CI)	P-value
^b^Pre-CCL2 (MCP-1)	0.802 (0.623–0.921)	< 0.001
Pre-GM-CSF	0.794 (0.614–0.916)	< 0.001
Pre-IL-17A	0.796 (0.616–0.917)	< 0.001
^c^Post-PD-L1	0.713 (0.526–0.858)	0.022
Combinations of two predictors		
Pre-CCL2 (MCP-1) + pre-GM-CSF	0.806 (0.628–0.924)	< 0.001
Pre-CCL2 (MCP-1) + pre-IL-17A	0.850 (0.680–0.951)	< 0.001
Pre-CCL2 (MCP-1) + post-PD-L1	0.822 (0.646–0.934)	< 0.001
Pre-GM-CSF + pre-IL-17A	0.798 (0.619–0.918)	< 0.001
Pre-GM-CSF + post-PD-L1	0.899 (0.740–0.977)	< 0.001
Pre-IL-17A + post-PD-L1	0.901 (0.743–0.978)	< 0.001
Combinations of three predictors		
Pre-CCL2 (MCP-1) + pre-GM-CSF + pre-IL-17A	0.846 (0.675–0.949)	< 0.001
Pre-CCL2 (MCP-1) + pre-GM-CSF + post-PD-L1	0.899 (0.740–0.977)	< 0.001
Pre-CCL2 (MCP-1) + pre-IL-17A + post-PD-L1	0.903 (0.745–0.979)	< 0.001
Pre-GM-CSF + pre-IL-17A + post-PD-L1	0.903 (0.745–0.979)	< 0.001
Combinations of all four predictors	0.899 (0.740–0.977)	< 0.001

^a^ Variables with P < 0.05 when applying univariate analysis of logistic regression are included as predictors. ^b^Pre-; pre-cerclage, ^c^Post-; post-cerclage.

AUC, area under the receiver operating characteristic curve; CI, confidence interval.

### 3.6 Correlations of ICRs and ICKs

The correlation of eleven variables, including pre-cerclage levels of CCL2 (MCP-1), CXCL10 (IP-10), GM-CSF, IL-1α, IL-6, IL-10, and IL-17A, and post-cerclage levels of LAG-3, CTLA-4, CD86 (B7-2), and PD-L1, was analyzed (**Supplementary Table 5**). Among these markers, the pre-cerclage markers were positively correlated with one another, and the post-cerclage markers demonstrated positive intercorrelations as well. Although most correlations between pre- and post-cerclage markers were negative, a notable exception was a positive correlation between pre-cerclage IL-17A and post-cerclage PD-L1 levels.

## 4 Discussion

In this study, we investigated the dynamic changes of ICRs and ICKs before and after cervical cerclage, and identified markers reflecting disease severity, such as bulging membranes, and those predictive of preterm birth. Significant differences in the immunologic microenvironments were observed before and after cerclage. Most ICKs sampled prior to cerclage were elevated in patients undergoing emergent cerclage with a bulging membrane, indicating a heightened local immune-inflammatory state. Among those, pre-cerclage IL-17A emerged as a significant predictor of preterm birth on multivariate analysis. Notably, the combination of pre-cerclage IL-17A and post-cerclage PD-L1 yielded an excellent predictive performance (AUC = 0.901).

The clinical benefits of cervical cerclage remain controversial (10, 18). While cerclage reduces the risk of recurrent preterm birth, it is associated with intraoperative risks, including rupture of membranes and cervical trauma due to its invasive nature (19, 20). Nonetheless, several studies have reported that cerclage can prevent ascending intra-amniotic infection and decrease local levels of proinflammatory cytokines (9, 11), thereby lowering rates of preterm birth and miscarriage. Our study expands this knowledge by simultaneously analyzing a comprehensive panel of 34 ICRs and ICKs, demonstrating dynamic immunologic changes following cervical cerclage and identifying potential biomarkers of disease severity and pregnancy outcome.

Using PCA and subsequent MANCOVA, we found marginal global changes in ICRs and ICKs, reflecting the limited cohort size due to the rarity of CI. Nonetheless, specific trends were evident: 12 out of 17 ICKs increased after cerclage, whereas several soluble ICRs decreased (**Table 1**). This reciprocal relationship aligns with the role of ICRs in suppressing immune activation. Reduced activity of ICRs permits T cell activation and subsequent upregulation of proinflammatory cytokines, including IFN-γ and IL-6, both of which are implicated in systemic inflammation (21, 22). In contrast, a prior study of 28 patients reported significant post-cerclage reductions in cervicovaginal IL-1β, IL-6, IL-8, MCP-1, and IFN-γ (11). The key methodological difference lies in the sampling method. Their follow-up specimens were obtained approximately one month later, whereas ours were collected 4.0 days (IQR: 3.0–7.0 days) postoperatively, before discharge (**Table 2**). Thus, our findings may represent the acute postoperative inflammatory surge, whereas the longer interval study might reveal subsequent resolution of the inflammatory status. Importantly, the short sampling interval after cerclage has clinical implications for real-world prediction of outcomes.

Our cohort included both urgent and emergent cerclage cases. Urgent cerclage was performed for cervical shortening detected on ultrasound in women without bulging membranes, whereas emergent cerclage was indicated for advanced dilation with bulging membranes (23–25). In the emergent cerclage group with bulging membranes, hospitalization was significantly longer, and pre-cerclage ICKs were markedly higher than those of the urgent cerclage group, consistent with a severe inflammatory status (**Table 3**). Additionally, among ICRs, TIM-3, HVEM, and CD40 levels in endocervical samples before cerclage were higher than those of the urgent cerclage group. The CD40-CD40L pathway, activated during uterine infections, is known to upregulate proinflammatory cytokines and has been linked to recurrent pregnancy loss (RPL) and IL-17 upregulation (26), supporting its potential role in cervical weakening and the promotion of preterm labor.

In post-cerclage samples, most ICKs showed no significant difference between bulging and non-bulging groups, with IL-1β even lower in the membrane bulging group than the non-bulging group (**Table 4**), suggesting that cerclage may mitigate excessive local inflammation, particularly in severe disease cases. Notably, ICRs were lower in patients with bulging membranes than in those with no bulging membranes, indicating persistent immune dysregulation.

Regarding pregnancy outcome, preterm birth was strongly associated with earlier gestational age at cerclage and the presence of bulging membranes, both markers of disease severity (27–29). Among the markers in pre-cerclage samples, ICKs rather than ICRs demonstrated significant associations with preterm birth, suggesting their potential as predictive biomarkers. In contrast, reduced ICRs from post-cerclage samples were more closely related to preterm birth than were ICK levels. Univariate analysis identified elevated pre-cerclage levels of CCL2 (MCP-1), GM-CSF, and IL-17A levels, and decreased post-cerclage PD-L1 levels as significantly associated with preterm birth.

These findings are immunologically plausible and supported by previous studies. CCL2 (MCP-1), a key chemokine recruiting macrophages, has been reported to be significantly elevated at the maternal-fetal interface in women experiencing preterm labor (30, 31). GM-CSF promotes macrophage infiltration in the cervix and uterus in mouse models of preterm birth, driving inflammation and tissue remodeling (32), while antibodies blocking GM-CSF can prevent preterm birth (33). IL-17A, produced by a range of innate immune cells in barrier tissues, plays a central role in early immune response. Although normally protective against pathogens, excessive IL-17A production during pregnancy, triggered by infections or *in-utero* inflammation, has been linked to adverse pregnancy outcomes, such as recurrent pregnancy losses, preterm birth, low birth weight, and heightened neonatal morbidity (34–36). In women with recurrent implantation failure with chronic endometritis, endometrial TGF-β, IL-10, IL-17, and autophagy are dysregulated (37). In chorioamnionitis, elevated IL-17A exacerbates fetal inflammatory responses, worsening fetal outcome (34, 35). Conversely, reduced PD-L1 expression at the maternal-fetal interface has been observed in spontaneous preterm labor, with both maternal stromal and fetal extravillous trophoblast cells showing lower PD-L1 levels compared with term pregnancies (38, 39). In women with RPL, decreased PD-1 and PD-L1 expressions on Th17 cells, as well as decreased PD-1 expression on Th1 cells, have been reported (38). These alterations may lead to enhanced Th1 and Th17 immunity, and a disrupted Th17/Th1/Treg balance, impairing maternal-fetal immune tolerance. Consequently, reduced PD-L1 expression may contribute to uncontrolled T cell activation and inflammatory responses at the maternal-fetal interface, thereby playing a pathogenic role in RPL and spontaneous preterm birth.

In ROC analysis, the predictability of IL-17A in our study (AUC = 0.796, 95% CI = 0.616–0.917) was comparable to the previous report (AUC = 0.715, 95% CI = 0.574–0.857) (35), validating its role as a meaningful biomarker. Importantly, the novel contribution of our study lies in the assessment of post-cerclage markers and combining them with pre-cerclage predictors to enhance prognostic performance. The combination of pre-cerclage IL-17A and post-cerclage PD-L1 demonstrated excellent discriminatory power (AUC of 0.901, 95% CI, 0.743–0.978), outperforming single markers. Adding additional predictors did not further improve accuracy, underscoring the efficiency and clinical practicality of this two-marker panel for identifying patients at high risk of preterm birth. Our results support the potential clinical utility of pre-IL-17A and post-PD-L1 for risk stratification, the groundwork for future translational studies and test development. Validation in larger, multicenter cohorts and the development of simplified, possibly point-of-care or ELISA-based assays would be required before these markers can guide individualized decisions such as intensified surveillance, adjunctive therapies, or timing of transfer to tertiary care.

The correlation analysis further reinforces the biological plausibility of this marker pair. While overall correlation between pre- and post-cerclage markers was negative, pre-cerclage IL-17A and post-cerclage PD-L1 exhibited a positive correlation (**Supplementary Table 5**), suggesting a coordinated immunologic shift that may reflect the interplay between proinflammatory activation and compensatory immune regulation. Together, these findings highlight a concise and mechanistically meaningful biomarker combination with high predictability, offering potential for early risk stratification and targeted clinical interventions in CI.

Previous excisional cervical procedures, such as cervical conization or loop electrosurgical excision procedure for HPV-associated cervical dysplasia, are recognized risk factors for cervical shortening, cervical insufficiency, and preterm birth (40, 41). In our cohort, none of the participants had a documented history of cervical conization or loop electrosurgical excision procedure, as confirmed by review of past operative, obstetric and gynecologic records. Therefore, these procedures were not included as covariates in the multivariable models.

The main limitation of our study is the relatively small sample size, which reflects the rarity of patients with CI. Nevertheless, the cohort size exceeded that of several previous studies in this field, lending credibility to the observed trends. Another limitation is the inability to evaluate membrane-bound forms of the investigated markers due to the lack of tissue samples. Soluble ICRs such as PD-L1, PD-L2, TIM-3, LAG-3, CTLA-4, and CD80 (CD86) are likely derived from a combination of activated T lymphocytes, antigen-presenting cells such as macrophages and dendritic cells, and possibly non-immune cells at the maternal–fetal interface, including cervical epithelial cells, stromal fibroblasts, and extravillous trophoblasts, which are known to express membrane-bound checkpoint molecules and can shed or release soluble forms (42, 43). Proinflammatory cytokines and chemokines such as IL-17A, IL-6, CCL2 (MCP-1), and GM-CSF reflect the activity of Th17 cells, innate lymphoid cells, neutrophils, macrophages, and other innate immune cells recruited to the cervix in response to mechanical stress or subclinical infection or inflammation. The decreases in several soluble ICRs after cerclage, together with increases in multiple ICKs, may represent a state of heightened effector inflammation that could contribute to persistent cervical remodeling and early parturition in high-risk patients (44, 45). Further studies combining soluble markers with tissue immunostaining of the endocervix to localize ICRs and ICKs expression to specific cell types, flow cytometry of cervical cells, and functional assays are necessary to confirm these mechanistic pathways. Further large-scale, multicenter studies are warranted to validate these findings and to comprehensively investigate both soluble and tissue-bound immune markers, which may further elucidate the underlying mechanisms and refine predictive models for preterm birth.

## 5 Conclusions

This study revealed dynamic immunologic changes in ICRs and ICKs before and after cerclage, providing valuable data on endocervical markers that reflect disease status and predict preterm birth in patients with CI. Most ICKs were elevated in emergent cases with bulging membranes, indicating a severe inflammatory state, whereas ICRs were generally reduced after cerclage, consistent with diminished immune regulation during the acute phase of inflammation. Pre-cerclage samples remain clinically important for initial risk stratification, while post-cerclage samples, particularly PD-L1, add predictive value when combined with pre-cerclage IL-17A. This two-marker panel demonstrated excellent predictive performance for preterm birth. Understanding the interplay between ICRs and ICKs provides a foundation for biomarker-driven strategies to improve the prediction and management of CI and preterm birth.

## Data Availability

The datasets generated and analyzed during the current study are available in the Figshare repository at https://doi.org/10.6084/m9.figshare.30280570.v1.
