# Supplementary material for: Dynamics of immune-checkpoint regulators and inflammatory cytokines and chemokines associated with preterm birth before and after cervical cerclage
Source: Front Immunol. 2026 Jun 23;17:1731111. doi: 10.3389/fimmu.2026.1731111 (PMC13337457; doi:10.3389/fimmu.2026.1731111)
Supplement: Supplementary file 1 [file DataSheet1.docx]

Supplementary Material

# Supplementary Figure


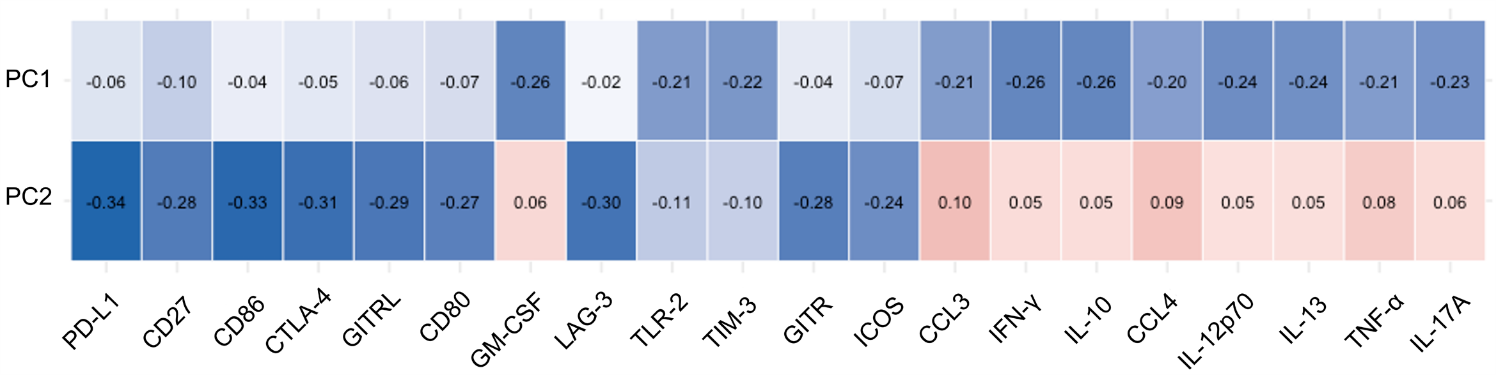


**Supplemental Figure S1**. Plot of multivariate analysis of covariance with principal component (PC) scores of PC1 and PC2 as dependent variables for the effects of cerclage and preterm birth.
